# Transcriptomic Profiling of Sea Buckthorn (*Hippophae rhamnoides* L.) Juice Intervention in 3D hAESC Spheroids: Implications for Cellular Plasticity and Tissue Regeneration

**DOI:** 10.3390/nu18193285

**Published:** 2026-10-07

**Authors:** Munkhzul Ganbold, Kazunori Sasaki, Kazutoshi Okawa, Makoto Kobayashi, Hiroko Isoda

**Affiliations:** 1 Alliance for Research on the Mediterranean and North Africa (ARENA), University of Tsukuba, Tsukuba 305-8572, Japan; 2 Open Innovation Laboratory for Food and Medicinal Resource Engineering (FoodMed-OIL), National Institute of Advanced Science and Technology (AIST), Tsukuba 305-8572, Japan; 3 Central Research Institute, ITO EN, Ltd., Shizuoka 421-0516, Japan; 4 Institute of Life and Environmental Sciences, University of Tsukuba, Tsukuba 305-8577, Japan

**Keywords:** sea buckthorn, *Hippophae rhamnoides*, human amniotic epithelial stem cells, spheroid cell culture, descriptive microarray

## Abstract

Background/Objective: Sea buckthorn contains an exceptionally rich profile of bioactive nutrients and metabolites, with established systemic therapeutic benefits across diverse pathological models. However, comprehensive data bridging these systemic outcomes with specific tissue responses are still lacking. This descriptive transcriptomic study establishes a predictive framework to guide the future application of sea buckthorn and its isolated derivatives in targeted preventive and therapeutic strategies. Methods: We utilized a 3D spheroid culture of normal human amniotic epithelial stem cells (hAESCs) to evaluate the bioactivity effects of sea buckthorn juice (SBJ) treatment. Results: Microarray analysis validated spheroid structural growth by revealing the upregulation of key upstream markers of proliferation. This SBJ-induced proliferation was supported by a coordinated upregulation of mitochondrial machinery, including oxidative phosphorylation, mitochondrial RNA processing, the TCA cycle, and mitochondrial membrane organization. Cell type signature analysis revealed that treated cells acquired highly proliferative and active extracellular matrix (ECM) remodeling traits. Furthermore, tissue database mapping showed that the cells shared strong transcriptional similarity with human embryo and Wharton’s jelly proliferative states. Muscle-type signatures, including cardiomyocyte, myoblast, and smooth muscle lineages, were consistently enriched. Conclusions: Overall, these findings provide a transcriptomic signature-based predictive model for the biological effects of SBJ. Based on gene ontology and tissue-specific enrichment profiles, our data offer hypothesis-generating predictions suggesting a potential role for SBJ in supporting cellular regeneration via stem cell proliferation. Furthermore, tissue-specific analysis indicates a putative capacity to modulate smooth muscle integrity, inferred from transcriptomic similarities with muscle-type gene signatures. While pure extracted compounds demonstrate beneficial effects, evaluating the whole SBJ matrix provides a broader understanding of its full, unique profile of bioactive properties. These in vitro findings highlight the potential value of the complete juice matrix, forming a predictive baseline for future in vivo and clinical evaluations.

## 1. Introduction

Sea buckthorn, *Hippophae rhamnoides* L., is a deciduous, nitrogen-fixing shrub known for its nutritional complexity and extreme environmental resilience. Characterized by its sharp thorns and dense cluster of yellow to orange berries, the plant naturally grows across the arid, mountainous, and coastal regions of Eurasia. In the early 1970s and 1980s, sea buckthorn was planted for protection and preservation of land due to its ability to develop a strong root system and to fix nitrogen and nutrients, and to resist low temperatures and drought conditions [1]. This intense ecological adaptation directly correlates with a concentrated pool of bioactive compounds in berries, seeds, and leaves [2,3]. Ethnopharmacological use of sea buckthorn has been known for over a thousand years. Historical applications focused on treating slow digestion, stomach malfunction, cardiovascular problems, liver injury, skin disorders, and mucosal ulcers [2,4]. In modern medicine, sea buckthorn plants are widely used in pharmaceutical, cosmetic, and nutraceutical industries for a diverse range of effects, and are produced in the form of drinks, oils, food supplements, and cosmetic products.

Sea buckthorn contains an exceptionally rich profile of bioactive nutrients and metabolites. Although the bioactive contents vary depending on the region, the berry has a high content of vitamins B, K, and C, which reach nearly 12 times higher than those found in oranges [5,6], carotenoids, a mixture of tocopherols, fatty acids including rare omega-7, omega-9, omega-6, and omega-3, flavonoids such as isorhamnetin and quercetin, phytosterols, and amino acids [5,7,8,9,10].

Sea buckthorn extracts showed antioxidant [11], anti-inflammatory, anti-microbial [12], and anti-viral [13] properties in different in vitro model systems [14]. Previous studies reported its protective effect against cardiovascular disease, hypertension, hyperlipidemia, and obesity by improving blood lipid profile and blood pressure, and by reducing platelet aggregation in animal models and human trials [15,16,17,18]. Studies on diabetes demonstrated that sea buckthorn seed residual extract reduced oxidative stress, serum glucose and triglyceride levels in diabetic rats [19], pulp oil augmented glucose-induced insulin secretion [20], and sea buckthorn juice reduced blood glucose and improved glucose tolerance in db/db mice [21,22]. A hepatoprotective effect was reported by increasing antioxidant markers and reducing liver damage enzymes in mice with CCl4-induced liver injury [23,24], and by reducing inflammatory cytokines (IL6, TNFα, NF-κB, and p65) in mice with LPS-induced liver injury [25]. Hepatic steatosis was mitigated by sea buckthorn polysaccharides through modulation of Nrf-2/HO1 signaling and gut microbiota [26]. Sea buckthorn oil accelerated the healing process of gastric ulcers and mucosal repair [27]. Application of sea buckthorn oil mixed with olive oil on skin burn wounds showed better re-epithelialization with continuous basement membrane [28]. Three months of sea buckthorn oil oral supplementation improved vaginal atrophy, and vaginal mucosal dryness and thinning in postmenopausal women [29]. Moreover, polyphenols from sea buckthorn enhanced antioxidant enzymes and reduced serum inflammatory cytokines, TNFα and IL6, by decreasing eNOS, ICAM1, and LOX1 in rat aorta with hyperlipidemia [30].

Accumulating evidence from previous studies has well established the systemic therapeutic benefits of sea buckthorn across a diverse range of pathological models. These systemic improvements are characteristically marked by the downregulation of atherogenic serum lipid profiles, the suppression of proinflammatory cytokines, and the favorable modulation of gut microbiota composition. Although sea buckthorn, characterized by a rich, multicomponent matrix of compounds, is expected to exert pleiotropic effects across multiple physiological systems, comprehensive data bridging these systemic outcomes with specific tissue responses are still lacking. In an effort to bridge this gap, the present study utilized transcriptomic microarray analysis to systematically explore specific biological pathways and localized molecular dynamics that are preferentially activated by sea buckthorn, while predicting its primary target tissue and cell types. Rather than evaluating endpoint therapeutic intervention, this descriptive transcriptomic study serves as a predictive framework to guide the future application of sea buckthorn and its isolated bioactive derivatives in targeted preventive and therapeutic strategies.

In this study, we utilized a three-dimensional (3D) spheroid culture of normal human amniotic epithelial stem cells (hAESCs) to evaluate the bioactivity effects of sea buckthorn juice (SBJ) treatment. Because hAESCs are isolated from discarded postpartum placenta, they circumvent the ethical concerns inherently associated with embryonic stem cells. Furthermore, hAESCs retain multilineage differentiation potential across all three germ layers and possess non-tumorigenic and immune privilege properties [31]. Culturing hAESCs as 3D spheroids further optimizes this system by enhancing stemness maintenance, establishing natural oxygen and nutrient gradients, and maximizing critical cell-to-cell and cell-to-extracellular matrix interactions that are characteristically absent in conventional 2D cultures [32,33]. These features allowed us to assess how SBJ modulates diverse cell and tissue lineages within a safe, physiologically normal cellular kinetic model.

## 2. Materials and Methods

### 2.1. Sea Buckthorn Juice Sample Preparation

Two samples of commercially available SBJ from distinct production lots (designated as Sample 1 and Sample 2) manufactured by GUAMARAL SAJI, Takarajima, Tsukuba, Japan, were provided by ITO EN Ltd., Shizuoka, Japan. Each sample was freeze-dried to obtain a powdered form. The resulting freeze-dried powders were dissolved in DMSO using ultrasonication, followed by filtration through a 0.22 μm membrane filter to prepare the stock solution. The prepared samples were subsequently subjected to HPLC analysis and used for cell treatment.

### 2.2. HPLC Analysis

Qualitative and quantitative analyses of quercetin and isorhamnetin in two freeze-dried SBJ powder samples were performed using high-performance liquid chromatography (HPLC), Shimadzu, Kyoto, Japan. Chromatographic separation was carried out on an Agilent SB-C18 column (4.6 × 250 mm, 3.5 μm particle size, Agilent Technologies, Tokyo, Japan), maintained at 40 °C. The mobile phase consisted of solvent A (0.1% formic acid in water, *v*/*v*) and solvent B (100% acetonitrile), applied under a gradient elution program. The gradient was linearly increased from 0% B to 100% B over 45 min. The flow rate was set at 1.0 mL/min, and the injection volume was 10 μL. Detection was performed at a wavelength of 254 nm. Identification of quercetin and isorhamnetin was achieved by comparing retention times with those of authentic standards. Quantification was conducted based on peak areas using calibration curves generated from external standards of each compound.

### 2.3. Cell Culture and Treatment

Human amniotic epithelial stem cells (hAESCs) were obtained from the Tsukuba Human Tissue Biobank Center (THB), University of Tsukuba Hospital [34]. Placental tissues were collected from donors undergoing cesarean section after obtaining written informed consent. Tissue collection and cell isolation were approved by the Ethical Review Committee of the University of Tsukuba Hospital (Approval No. H27-58, approval date: 10 July 2015). All experimental procedures for the current study were conducted exclusively using these pre-established, biobank-derived cells; no additional patient recruitment or prospective tissue collection was required. hAESCs derived from a single donor were utilized for all experiments. The procedures for hAESC culture have been described previously [35]. Briefly, hAESCs were cultured in a blended medium consisting of F12 and DMEM (1:1) supplemented with 10% fetal bovine serum (FBS), epidermal growth factor (EGF, 10 ng/mL), epinephrine (0.5 μg/mL), hydrocortisone (36 ng/mL), triiodo-L-thyronine (4 pg/mL), insulin–transferrin–selenium (ITS), and antibiotic–antimycotic, and the culture medium was replaced every 2–4 days. For 3D spheroid formation, hAESCs were seeded at a density of 1 × 10^6^ cells per well into Lipidure™ (NOF Corporation, Tokyo, Japan)-coated 3D culture plates (Elplasia™, Corning Inc., Oneonta, NY, USA) and cultured for 72 h. After 72 h of initial culture, the spheroids were treated with SBJ for 7 days, with medium replacement every 48 h. Cells were seeded into 3D culture plates according to the manufacturer’s technical specifications; each well features 554 microcavities, theoretically yielding approximately 554 uniform micro-spheroids per well. To overcome the biomass limitations of single-well micro-spheroid cultures and ensure robust RNA yields, all micro-spheroids within a given well were pooled at the conclusion of the treatment period prior to total RNA extraction. Each individual well was treated as an independent experimental run, representing an intra-donor biological replicate (*n* = 3 wells for the untreated control group). For the treated condition, to account for potential lot-to-lot variability, three independent wells were assigned to each of two distinct SBJ lots. Data from these two lots were pooled for downstream differential expression analysis, yielding a total of six independent intra-donor replicates for the treated group (*n* = 6). Consequently, the individual data points evaluated in the PCA and differential expression workflows represent these independent, well-level experimental replicates across their respective groups. For all experimental treatments, SBJ stock solutions were diluted in culture media yielding final DMSO concentrations of 0.02% (*v*/*v*) for the 10 μg/mL dose, 0.05% (*v*/*v*) for the 25 μg/mL dose, and 0.1% (*v*/*v*) for the 50 μg/mL dose. Control groups were maintained in the culture media without the addition of a matching DMSO vehicle, as all experimental conditions remained at or below the 0.1% threshold widely accepted as biologically inert [36]. Spheroid diameter and surface area were measured from microscopic photos using ImageJ 1.53a software.

### 2.4. Total RNA Extraction and DNA Microarray

Total RNA was extracted using Isogen reagent (Nippon Gene, Toyama, Japan) following the manufacturer’s instructions. The microarray experiment was performed using the GeneChip Whole Transcript Plus Reagent kit and Clariom S array human (#902917, Applied Biosystems, Singapore) following the GeneChip WT PLUS Reagent kit user guide (MAN0018137, Applied Biosystems, MA, USA). The array cartridges were stained and washed on GeneChip Fluidics Station 450 and scanned on a GeneChip Scanner 3000 7 G using the Affymetrix GeneChip Command Console (AGCC) (version 4.1.2.1567G, Affymetrix, Inc. Santa Clara, CA, USA) platform to obtain raw data for further analyses.

### 2.5. Microarray Analysis

Obtained raw data files were assessed for sample quality control and normalized using the robust multichip average (RMA) algorithm (http://www.affymetrix.com) on the Transcriptome Analysis Console (TAC) (version 4.0.2.15, Affymetrix Inc. Santa Clara, CA, USA). Differentially expressed genes (DEGs) were identified using an unadjusted *p*-value cutoff of < 0.05 and a fold change threshold of ±1.3. A complete list of the identified DEGs, including fold changes, nominal *p*-values, and FDR-adjusted *p*-values (*q*-values), is provided in Supplementary File S1. A limitation of this specific gene-level filtering is the omission of a false discovery rate (FDR) correction, which was bypassed to maximize sensitivity for candidate gene discovery in this exploratory phase. Consequently, downstream analyses including the over-representation analysis (ORA), protein–protein interaction (PPI) network construction, and kinase, tissue, and disease-enrichment analyses relied on these nominally significant DEG lists (*p* < 0.05). Because these thresholds were relaxed, these specific downstream investigations must be interpreted strictly as exploratory and hypothesis-generating. However, to ensure robust biological conclusions, this relaxed threshold was restricted solely to individual DEG identification. For downstream Gene Set Enrichment Analysis (GSEA), a stringent multiple-testing correction was maintained, and results were only considered significant at an FDR *q*-value < 0.25 and *p*-value < 0.05. To identify the common and core transcriptomic signatures induced by SBJ treatment rather than lot-specific variations, data from the two distinct SBJ lots were pooled together for the downstream differential expression analysis. This approach ensured that the enriched terms represent robust and reproducible biological effects consistent across different preparations.

Principal component analysis (PCA) and volcano plots were created using the TAC software (version 4.0.2.15, Affymetrix Inc. Santa Clara, CA, USA). Heatmaps were visualized on Morpheus (https://software.broadinstitute.org/morpheus, accessed on 18 July 2026). GSEA was conducted to identify enriched hallmarks, Reactome and KEGG pathways, gene ontology terms, and cell type signatures using the Human Molecular Signatures Database (MSigDB v2026.1) on desktop GSEA software (GSEA v4.4.0) [37,38]. Venn diagram data were analyzed using Venny 2.1. (https://bioinfogp.cnb.csic.es/tools/venny/index.html, accessed on 5 July 2026). Enrichment bubble plots were generated using SRplot (https://www.bioinformatics.com.cn/srplot, accessed on 6 July 2026) [39] using the GSEA enrichment results. Over-representation analysis (ORA) on gene ontologies, Reactome and KEGG was mapped using g:Profiler (https://biit.cs.ut.ee/gprofiler, accessed on 13 July 2026). A bubble network was created using Metascape (https://metascape.org/) [40]. General and tissue-specific protein–protein interaction (PPI) network analysis was performed on the online visual analytics platform NetworkAnalyst 3.0 (https://www.networkanalyst.ca/NetworkAnalyst/home.xhtml, accessed on 20 July 2026) [41]. Kinase enrichment analysis was conducted using the online Kinase Enrichment Analysis 3 (KEA3) tool (https://maayanlab.cloud/kea3/, accessed on 21 July 2026) [42]. ARCHS4 tissue enrichment was performed using the ARCHS4 database through the Enrichr online platform (https://maayanlab.cloud/Enrichr/, accessed on 7 July 2026) [40], and Human Protein Atlas (HPA) tissue-specific enrichment analysis by tissue-specific gene enrichment tool (https://tissueenrich.gdcb.iastate.edu/, accessed on 17 July 2026) [43]. Disease annotation analysis was performed on PANGEA using Alliance of Genome Resources (AGR) annotation (https://www.flyrnai.org/tools/pangea/web/, accessed on 21 July 2026).

### 2.6. Statistical Analysis

Spheroid quantification statistical analysis was conducted on GraphPad Prism (v.11.0.2) using one-way ANOVA followed by Dunnett’s multiple comparison test. Data were shown as mean ± SD with * *p*-value < 0.05 and *** *p*-value < 0.001. Statistical evaluation of microarray enrichment analyses was automated through the integrated functions of the utilized tools.

## 3. Results

### 3.1. Quercetin and Isorhamnetin Content in SBJ

**Figure 1:**
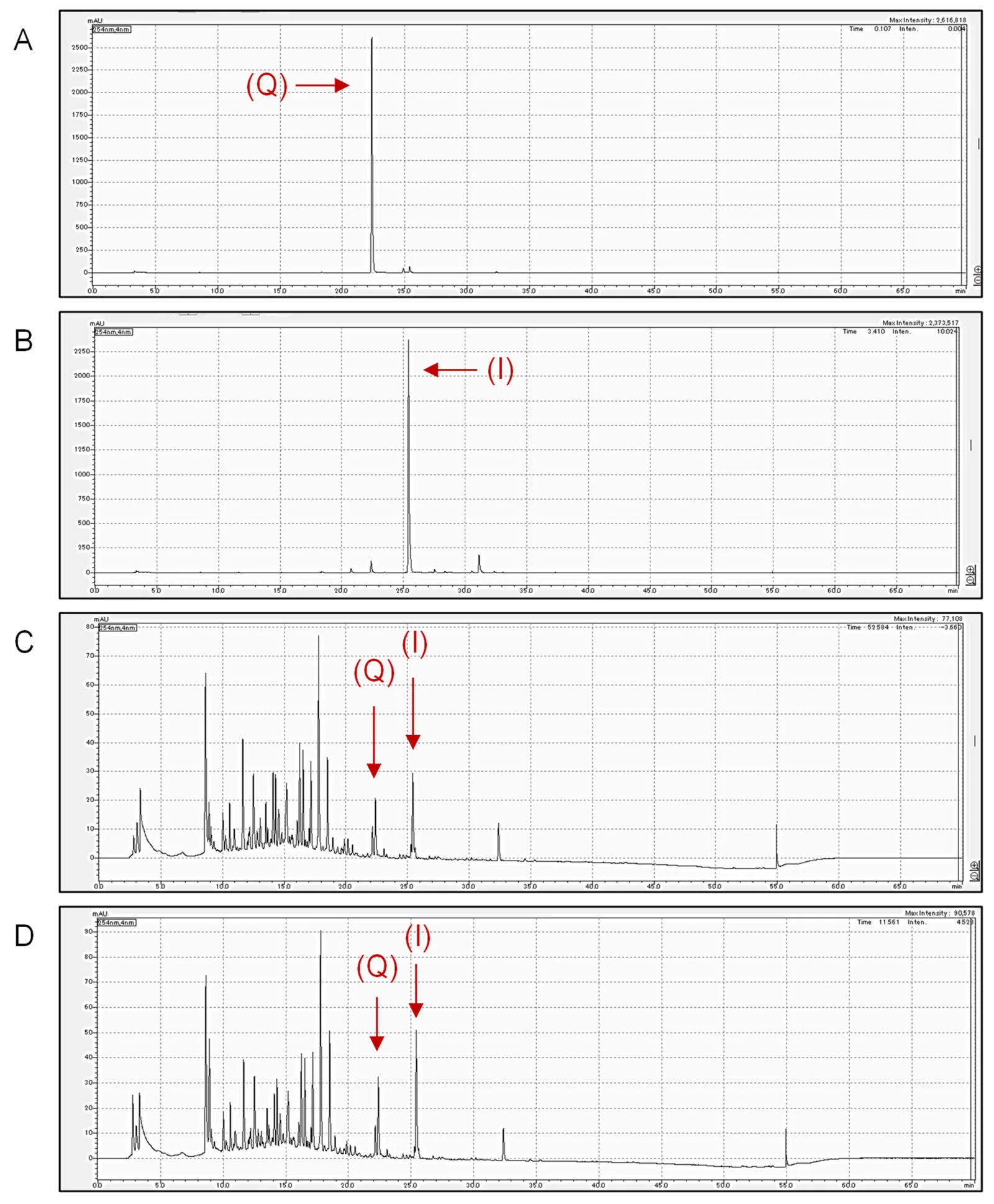
HPLC chromatograms of SBJ. (**A**) Quercetin standard, (**B**) isorhamnetin standard, (**C**) SBJ sample 1, and (**D**) SBJ sample 2. Q, quercetin, and I, isorhamnetin.

For the chemical characterization of the SBJ preparations, quercetin and isorhamnetin were selected as representative bioactive markers due to their well-documented abundance and potent antioxidant properties within the sea buckthorn matrix. For the quantification of quercetin and isorhamnetin, calibration curves were constructed using five standard concentrations (0.03125, 0.0625, 0.125, 0.25, and 0.5 mg/mL). The calibration curves exhibited excellent linearity, with a coefficient of determination (R^2^ = 0.997). As shown in the HPLC chromatograms (Figure 1), two SBJ samples exhibited distinct peaks that matched the retention times of the quercetin and isorhamnetin standards, confirming the presence of these compounds. HPLC-based quantification of quercetin and isorhamnetin in two SBJ samples showed that Sample 1 contained 0.102 ± 0.002 mg/g quercetin and 0.128 ± 0.015 mg/g isorhamnetin, whereas Sample 2 contained 0.196 ± 0.012 mg/g quercetin and 0.310 ± 0.003 mg/g isorhamnetin (*n* = 3).

### 3.2. Morphological and Transcriptomic Modulation of hAESCs Spheroid by SBJ

**Figure 2:**
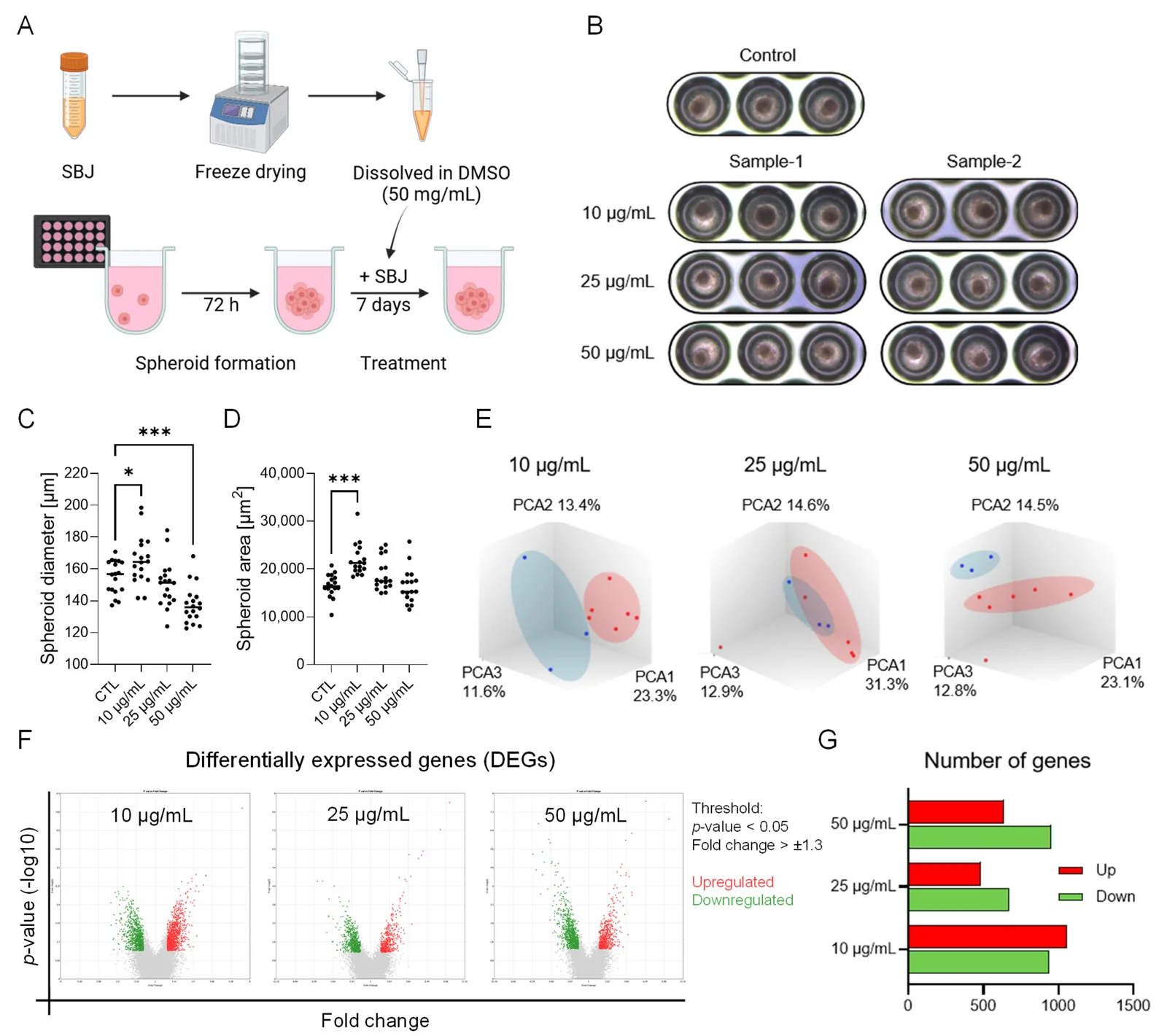
Differentially expressed genes of hAESC spheroid treated with SBJ. (**A**) Schematic diagram of SBJ sample preparation and spheroid treatment design. (**B**) Brightfield microscopic images of spheroid formation. Microwell diameter: 400 µm. (**C**) Spheroid diameter and (**D**) surface area quantified from microscopic images. * *p*-value < 0.05, and *** *p*-value < 0.001. (**E**) Principal component analysis (PCA) showing clusters of control (blue) and SBJ-treated (red) spheroids. (**F**) Volcano plot analysis of DEGs. (**G**) Number of up- and downregulated DEGs.

To evaluate the regulatory effect of SBJ on 3D stem cell growth, human amniotic epithelial stem cells (hAESCs) were cultured for 72 h to form spheres and subsequently treated with 10, 25, and 50 µg/mL of SBJ for 7 days (Figure 2A). The treatment dose was determined based on the efficacy and safety profiles reported in previous studies [24,44,45]. Spheroid morphology was evaluated by measuring the diameter and the surface area of aggregates. Following 7 days of treatment, geometric profiling confirmed that the cells successfully formed and maintained a consistent and spherical structure in all groups (Figure 2B). The spheroid diameter and surface area in the 10 µg/mL treated group were significantly higher than those of the control group (Figure 2C,D). In contrast, increasing the concentration to 50 µg/mL resulted in a significantly lower spheroid diameter compared to the control. Notably, while the higher concentrations impacted diameter, the 25 and 50 µg/mL treatment groups did not show any significant differences in surface area relative to the control group.

Next, to investigate the molecular alteration induced by the treatment of SBJ, a microarray analysis was conducted. Principal component analysis (PCA) demonstrated distinct clustering based on treatment groups, indicating differential gene expression profiles (Figure 2E). To identify specific transcriptomic variations, differentially expressed genes (DEGs) were isolated based on a threshold of fold change > ±1.3 and *p*-value < 0.05 (Figure 2F). Compared to the untreated control, the 10 µg/mL treated group had a total of 2000 DEGs, consisting of 1060 upregulated and 940 downregulated, whereas 25 µg/mL and 50 µg/mL exhibited 1152 and 1589 DEGs, consisting of 483/669 and 637/952 up/downregulated genes, respectively (Figure 2G). To isolate the core molecular signatures that are common or unique across the treatment groups, we next evaluated overlapping DEGs and treatment dose-associated hallmarks.

### 3.3. Identification of Overlapping DEGs and Enriched Biological Hallmarks

**Figure 3:**
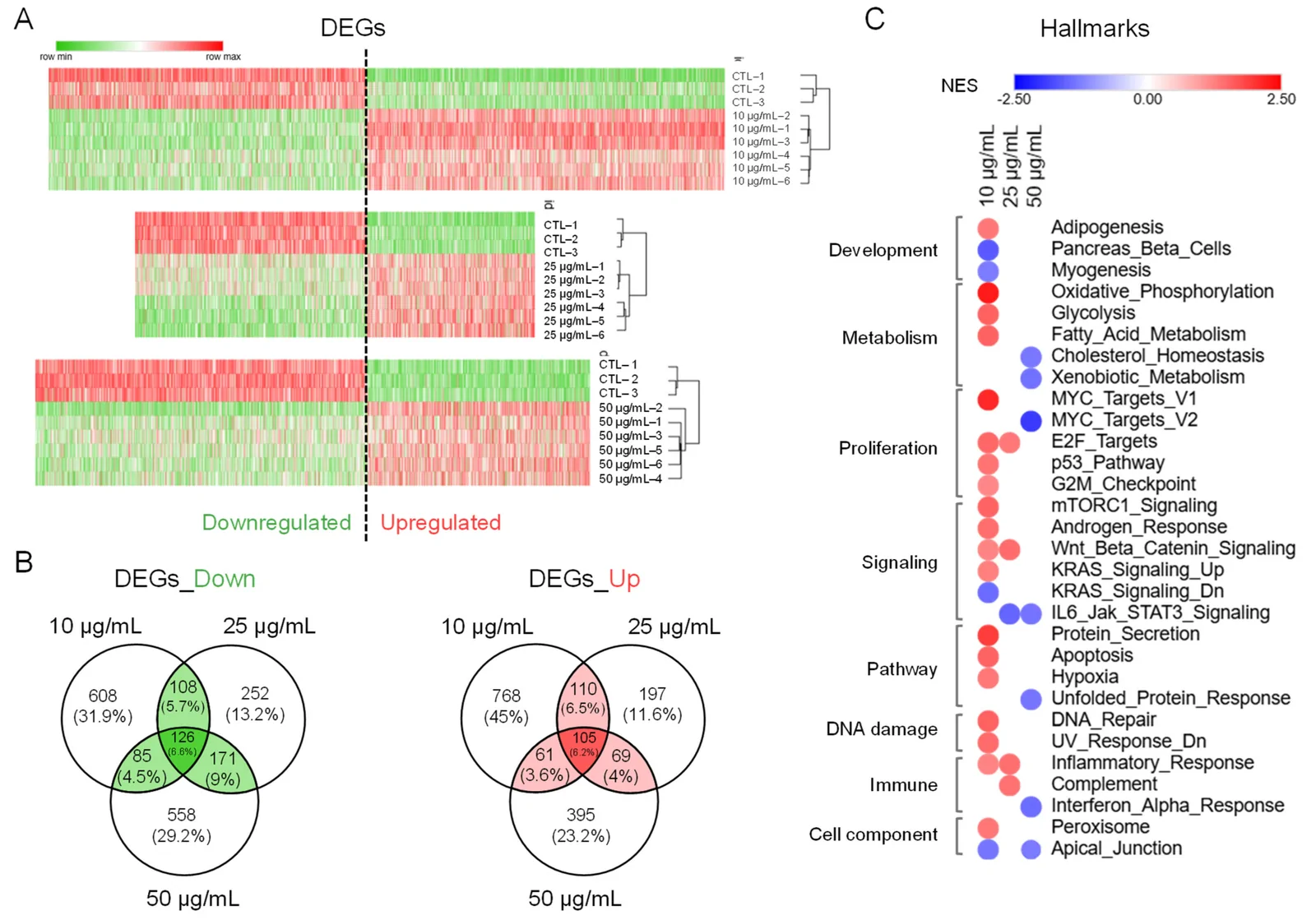
DEGs and hallmark enrichment analysis. (**A**) Heatmap of up- and downregulated DEGs. (**B**) Venn diagram of downregulated (**left**) and upregulated (**right**) DEGs from 10, 25, and 50 µg/mL treated groups. (**C**) Heatmap of hallmarks enriched in each treatment group. GSEA FDR *q* < 0.25 and *p* < 0.05.

To characterize the global expression patterns across the different dose treatment conditions, hierarchical clustering analysis of the DEGs was performed and demonstrated as a heatmap (Figure 3A). The heatmap revealed a distinct relationship between treatment dose and transcriptional regulation. Specifically, while there is a progressive decline in the number of upregulated genes as the treatment dose increases, the ratio of downregulated to upregulated DEGs expanded from 0.9 to 1.5 in a dose-dependent manner. This transition suggests a shift from targeted gene activation at lower doses to widespread transcriptional repression at higher doses. Moreover, Venn diagram analysis highlighted minimal transcriptomic overlap across the treatment conditions. Among all treated groups, 126 genes representing 6.6% of the total were commonly downregulated, while 105 genes (6.2%) were commonly upregulated (Figure 3B). The highest percentage of unique DEGs was observed in the 10 µg/mL treated group, containing 45% upregulated and 32% downregulated genes. This overlap shows that the majority of DEGs were unique to specific treatment groups, specifically in 10 µg/mL, suggesting distinct and dose-specific molecular mechanisms.

To elucidate functional biological pathways associated with the observed transcriptomic shifts, gene enrichment analysis of hallmarks was performed across the treatment groups (Figure 3C). The most significantly enriched hallmarks (*p*-value < 0.05 and FDR *q*-value < 0.25) were predominantly observed in the 10 µg/mL treated group. Specifically, upregulated hallmarks were related to metabolic processes, cell proliferation networks and cell stress pathways, including apoptosis, hypoxia, and DNA repair, while developmental hallmarks were downregulated. In contrast to this extensive transcriptomic alteration at the lower dose, the 25 and 50 µg/mL treated groups exhibited a minimal overall hallmark enrichment. Notably, significant pathway enrichment at 50 µg/mL was limited to the downregulated hallmarks, cholesterol homeostasis and MYC targets V2. Collectively, these findings highlight that 10 µg/mL SBJ triggers activation of molecular pathways coordinating cell proliferation, adaptive stress responses, and metabolic activation.

### 3.4. SBJ Activates Transcriptomic Signatures in Oxidative Phosphorylation and Proliferation

**Figure 4:**
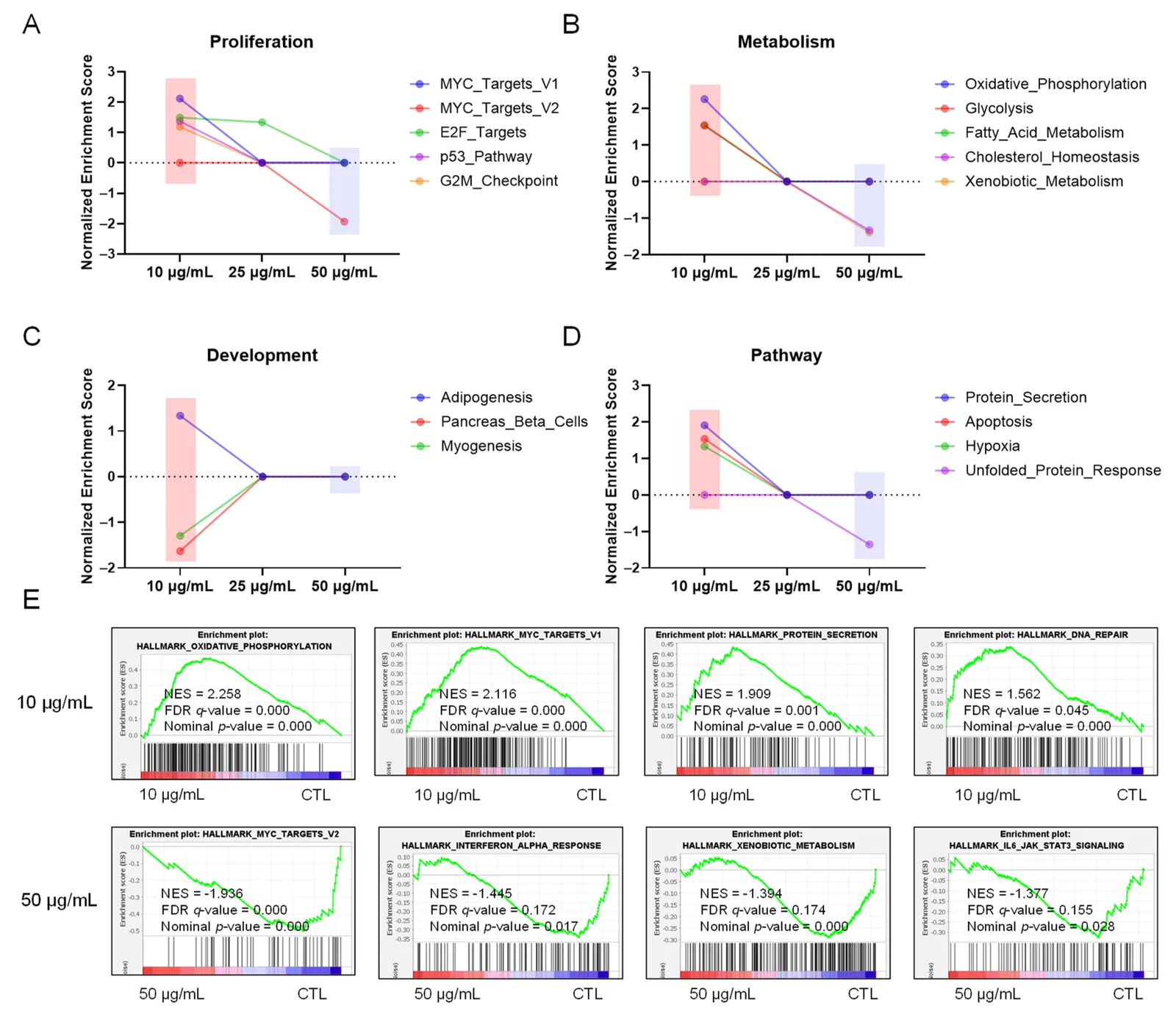
Comparison of enriched hallmarks. Comparison of normalized enrichment scores of 10, 25, and 50 µg/mL treated groups in the (**A**) Proliferation, (**B**) Metabolism, (**C**) Development, and (**D**) Pathway process categories. (**E**) Top enriched hallmarks in 10 and 50 µg/mL treated groups.

To precisely map the directional transcriptomic shift induced by increasing doses, a comparison of most enriched hallmarks related to proliferation, metabolism, development, and pathway was conducted by its normalized enrichment score among treatment groups (Figure 4A). Gene sets in MYC targets are essential downstream effectors in cell cycle and related pathways for ribosome biogenesis and protein synthesis. E2F targets are required for G1/S phase transition and DNA replication during cell cycle progression. These gene sets were found to be upregulated in the 10 µg/mL group, while increasing doses tend to repress this activation. In addition to decreasing tendency of E2F targets activation, MYC targets V2 hallmark consisting of more specific and strongly regulated genes than MYC targets V1 was significantly downregulated in 50 µg/mL treated group, suggesting higher dose of treatment compromise the intrinsic activation of cell proliferation.

Metabolism-related hallmarks were highly activated in the 10 µg/mL group, directly corresponding to the concurrent activation of cell proliferation markers, while the 25 and 50 µg/mL groups showed no difference compared to the control (Figure 4B). The oxidative phosphorylation pathway represents mitochondrial ATP production through the electron transport chain. Upregulation of this hallmark indicates elevated energetic demand and production in cells treated with SBJ, more prominently with the lower dose. At the higher dose, transcriptomic profiling revealed a downregulation of pathways associated with xenobiotic metabolism and cholesterol homeostasis. These signatures suggest a potential vulnerability in the cellular defense system and membrane maintenance mechanism under higher-dose treatment conditions. Furthermore, these differential expression profiles predict that cells treated with the lower dose of SBJ may shift toward aerobic respiration while maintaining an active glycolytic pathway.

Lineage-specific hallmarks were enriched in three developmental pathways: adipogenesis, pancreatic beta cells, and myogenesis pathways. In the 10 µg/mL group, pancreatic beta cells and myogenesis were markedly downregulated, and adipogenesis was upregulated, while higher-dose-treated groups failed to show any difference compared to the control (Figure 4C). Furthermore, pathways related to apoptosis, hypoxia, and protein secretion were enriched in the 10 µg/mL group, while the unfolded protein response was downregulated in the 50 µg/mL group (Figure 4D). These findings showed that cellular stress adaptation and secretory pathways were preserved in the lower-dose treatment, while a critical protein quality control system was compromised at the higher dose. Moreover, direct comparison of the most enriched hallmarks between the 10 and 50 µg/mL groups demonstrated a clear, contrasting difference in overall cell behavior in response to differential doses (Figure 4E). This comparative profiling revealed that 10 µg/mL treatment stimulated an advantageous phenotypic state with high proliferation and metabolic activation accompanied by regulated stress adaptation, while 50 µg/mL triggered a reversal, downregulating core proliferative pathways and the cell defense response.

### 3.5. SBJ Drives hAESCs to Highly Proliferative and Metabolically Active State

**Figure 5:**
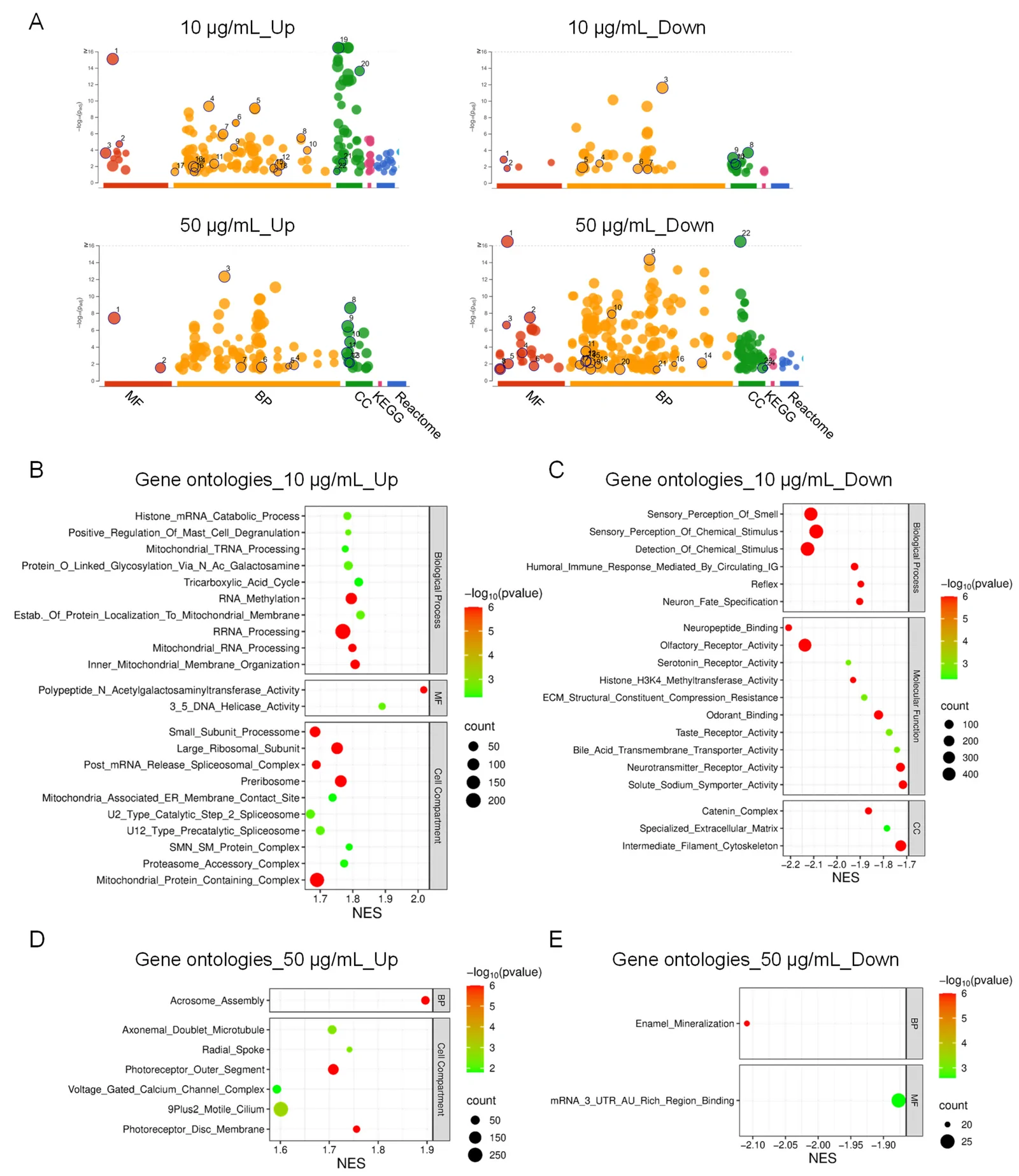
Gene ontology analysis on 10 and 50 µg/mL treated groups. (**A**) Functional profiling map of DEGs on molecular function (MF), biological process (BP), cell compartment (CC), KEGG and Reactome pathways. Top enriched (**B**) upregulated in the 10 µg/mL treated group, (**C**) downregulated in the 10 µg/mL treated group, (**D**) upregulated in the 50 µg/mL treated group, and (**E**) downregulated in the 50 µg/mL treated group gene ontologies. GSEA FDR *q* < 0.25 and *p* < 0.05.

To examine the functional differences in dose response, we directly compared gene ontologies between up- and downregulated genes (*p* < 0.05 and fold change > ±1.3) from 10 and 50 µg/mL using over-representation analysis. Functional enriched landscape showed more expanded and diversified array in upregulated genes in 10 µg/mL group (Figure 5A). This lower concentration triggered multiple biological processes and molecular functions, indicating highly activated cellular programming. Conversely, the 50 µg/mL group exhibited dominant accumulation in downregulated functional processes compared to that of the 10 µg/mL group, while its upregulated gene array remained lower and scarce. This macro-level profiling confirms that the lower dose induced complex and multiple biological processes, whereas the higher dose exerted widespread functional repression.

Subsequently, we conducted GSEA of gene ontology (GO) terms across both dose groups to determine precise phenotypic clusters, and the top 10 GO terms (*p* < 0.05 and FDR *q* < 0.25) were selected. Within the upregulated GO Biological Process of 10 µg/mL, dominant clusters were concentrated on translational machinery, including rRNA processing, mitochondrial RNA processing, and mitochondrial tRNA, and energetic and structural adaptation, evidenced by activation of inner mitochondrial membrane organization, the tricarboxylic acid (TCA) cycle, and the histone mRNA catabolic process (Figure 5B). Consistent with this transcriptional activation, structural complex activation in cell compartments, and enzymatic reactions in GO such as 3′,5′-DNA helicase activity showing active DNA replication in molecular function highlight cellular commitment to biomass accumulation, mitochondrial biogenesis, and structural remodeling. Moreover, the downregulated functional profile in 10 µg/mL showed biological processes of sensory perception of smell, detection of chemical stimulus, and olfactory receptor activity, indicating that the spheroids deactivated sensory machinery to prioritize energy for active proliferation and spheroid expansion (Figure 5C).

In contrast to the expanded transcriptomic remodeling observed in 10 µg/mL, the 50 µg/mL treated group showed a markedly decreased amount of GO enrichment in up- and downregulated gene sets. Significantly upregulated GO terms were acrosome assembly in the biological process, axonemal doublet microtubule and radial spoke in the cell compartment, and no enrichment in molecular function (Figure 5D). Downregulated terms were restricted to enamel mineralization, and mRNA 3′-UTR AU-rich region binding, which indicates a disruption in mRNA stability and the post-transcriptional mechanism (Figure 5E). These findings indicate that 10 µg/mL is optimized for a transcriptional profile with active biomass accumulation and physical expansion by upregulation of proliferative networks and mitochondrial respiration machinery, whereas 50 µg/mL collapses these active networks.

### 3.6. Network Mapping and Pathway Architecture

**Figure 6:**
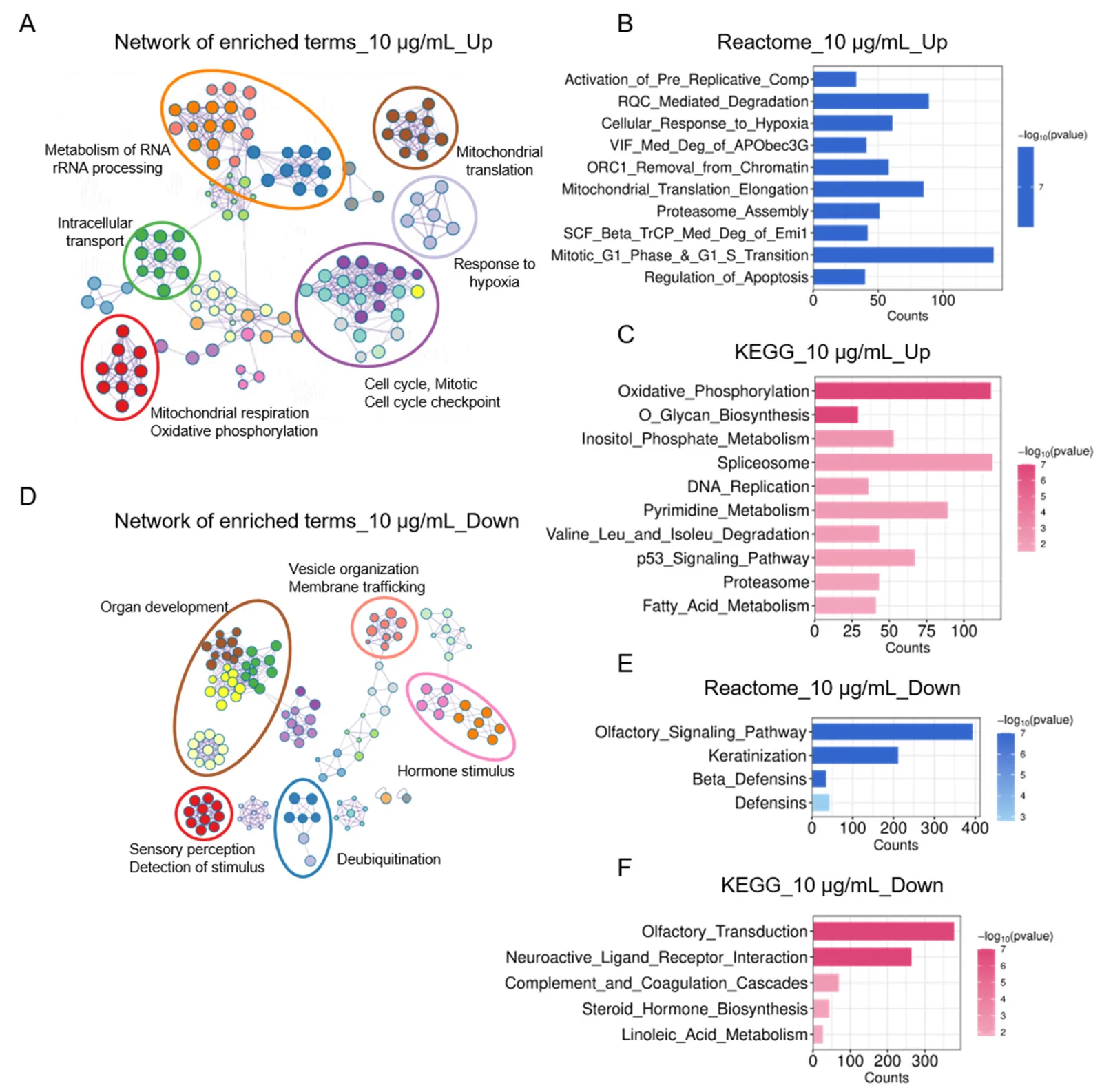
Network of enriched terms and Reactome and KEGG pathways. (**A**) Network of enriched terms in 10 µg/mL treated group upregulated DEGs. (**B**) Reactome and (**C**) KEGG pathways upregulated in the 10 µg/mL treated group. (**D**) Network of enriched terms in the 10 µg/mL treated group downregulated DEGs. (**E**) Reactome and (**F**) KEGG pathways downregulated in the 10 µg/mL treated group. GSEA FDR *q* < 0.25 and *p* < 0.05.

To elucidate the interconnected topological relationship among the enriched terms, we mapped the upregulated and downregulated functional networks for the 10 µg/mL group. Upregulated network mapping revealed primary functional clusters in RNA processing, mitochondrial translation and respiration, mitotic cell cycle, intracellular transport, and response to hypoxia (Figure 6A). Beyond the individual mapped hubs, in fact, these terms are highly interconnected and coordinated cellular responses. RNA processing indicates upregulation of protein synthesis required for mitotic cell division and DNA replication, coinciding with mitochondrial translation for oxidative phosphorylation to meet the high energetic demand. Newly synthesized molecules were distributed across the cell compartments by vesicular trafficking and protein secretion (intracellular transport). In addition, within the context of growing a spheroid structure, low oxygen adaptation pathways reflect the healthy homeostatic metabolic response as a survival mechanism (response to hypoxia). To validate these network clusters, we also conducted GSEA Reactome and KEGG pathway enrichment analysis. Ribosome quality control-mediated degradation (Reactome), inositol phosphate metabolism, spliceosome, and pyrimidine metabolism (KEGG) strongly corroborated the RNA processing cluster (Figure 6B,C). The active metabolic pathway was validated by enrichment in oxidative phosphorylation (KEGG) and mitochondrial translation elongation (Reactome). DNA replication (KEGG), activation of the pre-replicative complex, and ORC1 removal from chromatin (Reactome) overlap support the activation of replication origin and G1/S phase progression.

Next, network analysis was performed on 10 µg/mL downregulated gene sets to identify which biological processes and pathways were most significantly repressed. The resulting topology revealed clusters of sensory perception, organ development, hormone stimulus, deubiquitination, and vesicle organization (Figure 6D). The dominantly enriched cluster was downregulation of baseline sensory machinery and sensitivity to external hormonal activation, which was directly validated by significant enrichment of olfactory transduction and neuroactive ligand–receptor interaction (KEGG) and olfactory signaling pathway (Reactome) (Figure 6E,F). This downregulated sensitivity to external stimuli directly reflects a transcriptional shift required for active spheroid expansion. Moreover, downregulated multilineage differentiation pathways corroborated keratinization and beta defensins (Reactome) pathways, indicating that the treated cells are delaying differentiation programming by prioritizing a highly plastic and proliferative state. This profile suggests a model where cells are predicted to redirect all available resources toward cell division and spheroid expansion. This potential metabolic shift is characterized by the upregulation of protein recycling pathways (deubiquitination) and the downregulation of membrane trafficking, which may serve to optimize intracellular transport.

### 3.7. Protein–Protein Interaction (PPI) Network and Tissue-Specific Characterization

**Figure 7:**
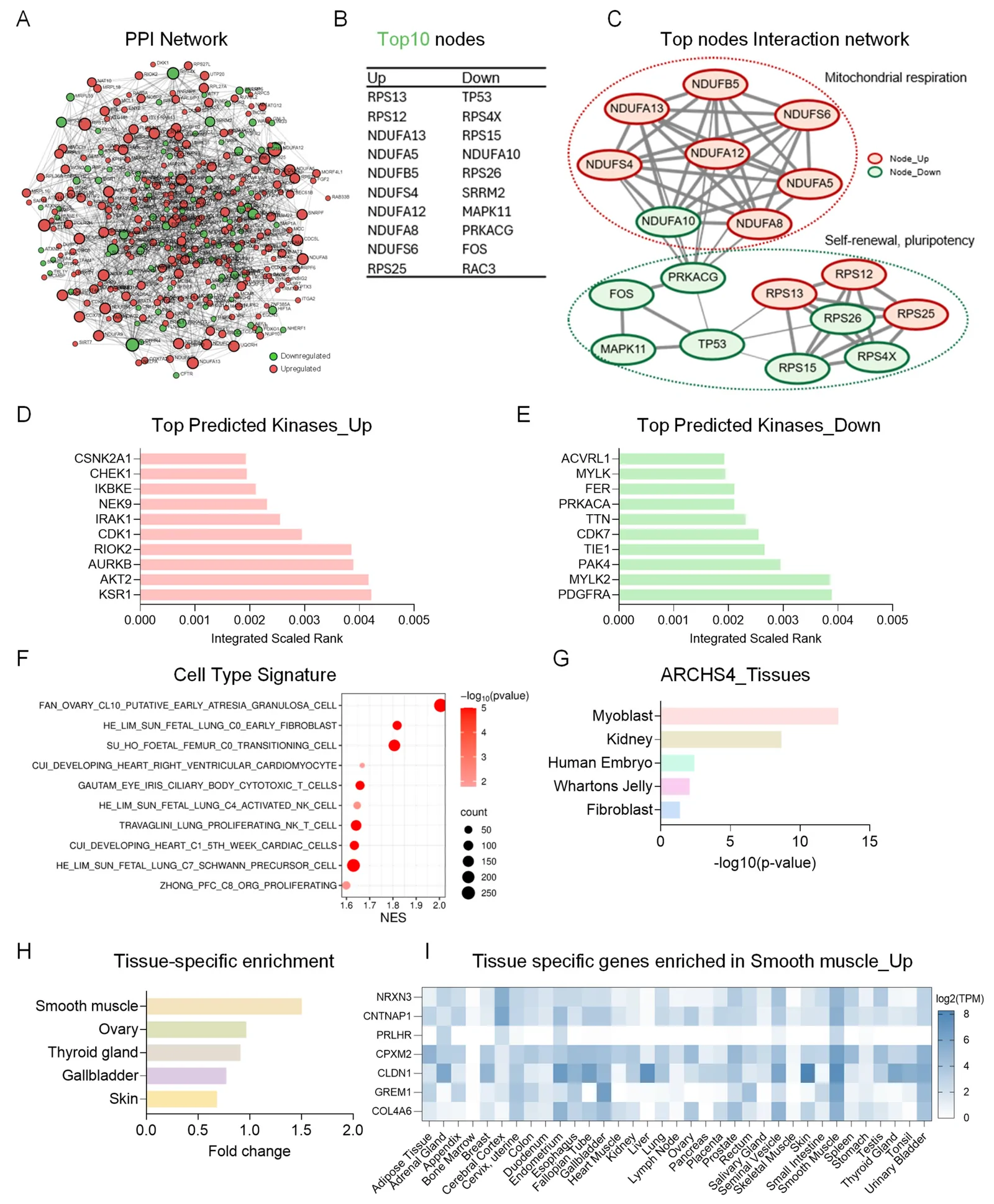
Protein–protein interaction (PPI) network analysis and cell type and tissue type signatures of the 10 µg/mL treated group. (**A**) PPI network of upregulated (red nodes) and downregulated (green nodes) genes. (**B**) List of top 10 upregulated and downregulated nodes from the PPI network. (**C**) Top10 nodes interaction network. Top (**D**) upregulated and (**E**) downregulated predicted kinases. (**F**) GSEA cell type signature. (**G**) ARCHS4 tissue enrichment and (**H**) Top5 HPA tissue-specific enrichment. (**I**) Heatmap of tissue-specific genes enriched in smooth muscle.

Next, we sought to explore the predicted functional interactions among DEGs with a PPI network analysis using both 10 µg/mL up- and down-regulated gene sets (Figure 7A). The highest-degree topological nodes representing the most connected central proteins within up- and downregulated networks were identified and shown in Figure 7B. We subsequently analyzed the topological connections of these top nodes selected from up- and downregulation to evaluate whether they are mutually interrelated and whether they are mapped into specific biological processes. Interestingly, the most upregulated nodes were highly interrelated and collectively corresponded to the activated mitochondrial respiration cluster (Figure 7C). Downregulated nodes were major regulators associated with cell cycle arrest and apoptosis (TP53, MAPK11, and FOS), and an effector for differentiation signaling (PRKACG). Concurrent downregulation of these nodes in addition to ribosomal subunit activation (RPS12, 13, 25) predicted to attenuate growth arrest signals and stress sensors. This coordinated transcriptomic profile points toward a potential shift toward a more proliferative and plastic cellular state, offering a compelling hypothesis for further functional investigation.

Furthermore, to complement the observed transcriptomic shift, we investigated the predicted kinases responsible for modulating DEG sets. The top predicted kinases regulating activated gene sets were highly correlated with cell cycle regulators and mitotic checkpoint kinases, and cell survival signaling (Figure 7D). CSNK2A1, CHEK1, NEK9, CDK1, and AURKB are known to be critical in mitotic phase and G2/M transition [46,47]. In addition, IKBKE, IRAK1, and RIOK2 play a key role in ribosome biogenesis [48,49]. Conversely, top kinases predicted from downregulated DEGs were associated with growth arrest, microenvironmental sensing, and differentiation pathways (Figure 7E). By downregulation of ACVRL1, TIE1, and PDGFRA kinases, cells exhibit a decreased number of surface receptors coupled with compromised intracellular remodeling (MYLK and MYLK2). Together with the downregulation of PRKACA, which triggers cell differentiation, the collective suppression of this kinase network indicates a restricted differentiation pathway, thereby sustaining cells in an active self-renewal state.

Next, to explore whether the SBJ-induced transcriptomic shift led the cells into any specific lineage states, we analyzed the data using the GSEA cell type signature database (Figure 7F). The top enriched cell types exhibited common characteristics based on their developmental origin and functional properties. Fetal lung, femur, and heart proliferating cell type signatures indicate that the cells are still in a state that requires high mitotic capacities to increase biomass and lack terminal differentiation features. Moreover, granulosa-type cells and early fibroblast and transitioning cells share a common feature in remodeling of ECM and cell junctions, suggesting a more mesodermal cell lineage. These cell type behaviors suggest that the cells in spheroid structure after SBJ treatment may retain an early embryonic-like, and metabolically and energetically resilient state, rather than initiating terminal specialized differentiation pathways.

To complement the single-cell type signature findings, we sought to conduct tissue-specific enrichment analysis on 10 µg/mL group upregulated DEGs using the ARCHS4 tissues database. The most enriched tissues were myoblast, kidney, human embryo, Wharton’s jelly, and fibroblast (Figure 7G). Consistent with the findings in cell type signatures, human embryo tissue shares common capabilities of mitotic potential and biomass production with fetal-stage proliferating cells. Wharton’s jelly and fibroblast tissue represent cellular behavior in building a physical scaffolding matrix and preserving plasticity. Myoblast and kidney tissue cells have developed a skeletal filament system required for cell movement and rearrangement. These characteristics suggest that the SBJ-treated hAESCs growing in spheroids undergo a phenotypic shift, allowing the cells to expand flexibly in a 3D structure. Furthermore, to complement our findings of tissue-specific signatures, we subjected upregulated DEGs from the 10 µg/mL group to analysis of organ-level tissue enrichment using the Human Protein Atlas (HPA). The most enriched tissue signatures were related to smooth muscle, ovary, thyroid gland, gallbladder, and skin (Figure 7H). Smooth muscle and gallbladder cells are specialized for mechanical stretch and contraction, thus having highly developed cytoskeletal structures. The activation of these pathways corroborates the structural flexibility observed in the expansion of 3D spheroids without stiff mechanical resistance. Interestingly, several genes enriched in the smooth muscle subset from the HPA overlapped with other top enriched tissue types (Figure 7I). These integrated network and tissue-specific analyses provide a model suggesting that the SBJ might exert its effects on tissue plasticity and structural adaptation. Specifically, the transcriptomic data point toward potential beneficial effects on smooth muscle, skeletal muscle, lung, and ovarian cell lineages, hypothetically driven by the modulation of self-renewal programs and metabolic flexibility.

### 3.8. Tissue-Specific PPI Network Analysis Across Enriched Lineages and Disease-Related Annotation

**Figure 8:**
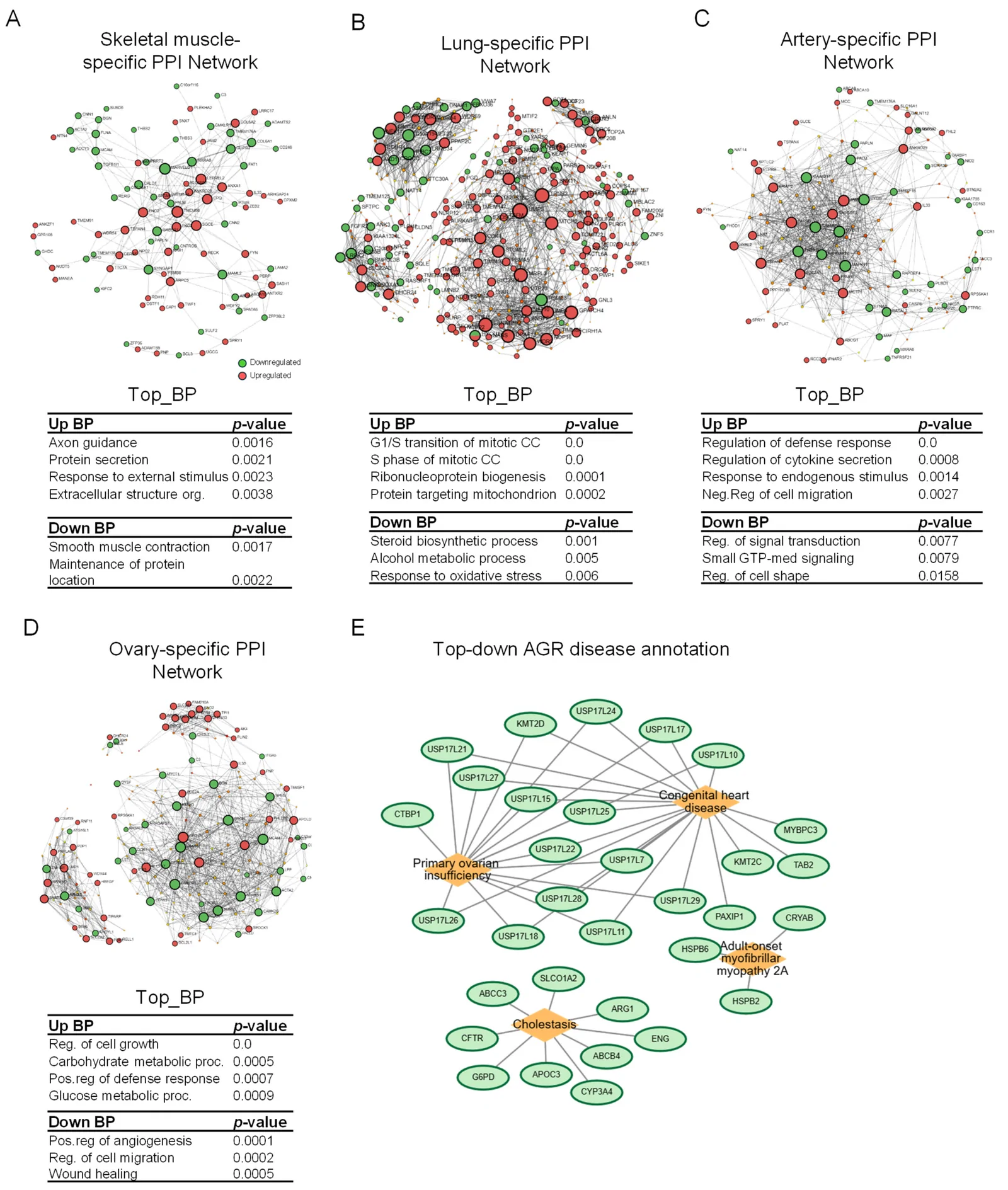
Tissue-specific PPI network and related top enriched biological process. PPI network for (**A**) skeletal muscle, (**B**) lung, (**C**) artery, and (**D**) ovary, and related up- and downregulated BPs. (**E**) Top enriched AGR disease annotation in downregulated DEGs of the 10 µg/mL treated group. The network of disease annotation (yellow) and related downregulated genes (green) is shown.

Based on the signatures identified through cell-type and tissue-type enrichment, we next sought to resolve the predicted intracellular signaling pathways associated with these respective tissue signatures. After mapping highly connected nodes, we clustered the most upregulated and downregulated biological processes modulated by the SBJ treatment. Within the skeletal muscle-specific PPI network, interconnected and upregulated protein nodes were associated with structural remodeling (protein secretion and response to external stimulus), directional expansion (axon guidance), and extracellular signaling (extracellular structure organization) (Figure 8A). Conversely, the downregulated protein network was enriched in structural contraction and protein retention, suggesting that the predicted effect of the SBJ could be to promote muscle cell regeneration and protection by activating extracellular matrix remodeling, active secretion, and directional expansion. Following muscle tissue, we analyzed a lung-specific PPI network using up- and downregulated DEGs. Upregulated protein nodes were highly concentrated in cell cycle and translation processes, while downregulation was found in non-essential metabolic pathways (Figure 8B). These transcriptomic alignments generate the hypothesis that SBJ may possess a putative role in counteracting pulmonary diseases characterized by disrupted tissue homeostasis, acting through the predicted stimulation of lung cell proliferation and tissue renewal pathways. Furthermore, following the smooth muscle and myoblast signatures enriched in tissue-specific analyses, we identified the PPI in the artery-specific protein network (Figure 8C). Biological processes among upregulated nodes are localized around the cell protection (regulation of defense response) and structural containment (negative regulation of cell migration) protein networks. In contrast, intracellular signal transmission and structural remodeling-related processes were downregulated. This simultaneous upregulation of localized defensive cytokines and reduced migratory mechanisms points to a highly specialized cellular state in vascular-type matrices, suggesting that the SBJ treatment might exert a protective influence on the vascular network by ensuring highly controlled smooth muscle type structural integrity. Lastly, we investigated the significant enrichment in the ovarian microenvironment. Generally, cell growth and metabolic processes were activated, while vessel development and cell motility-related transcriptomic signatures were compromised (Figure 8D). These enriched processes suggest a transcriptomic profile consistent with SBJ-induced activation of controlled cell growth coupled with glycolytic energy metabolism in ovarian-type signatures, rather than signatures associated with blood vessel formation and tissue motility.

We finally analyzed whether there was any clinical relevance between downregulated DEGs and established clinical phenotypes using the Alliance of Genome Resources (AGR) disease annotation database. Interestingly, significant enrichment of downregulated genes linked to primary ovarian insufficiency characterized by follicular depletion and a premature ovarian aging process reflects a potential protective and preventive effect of SBJ treatment (Figure 8E). Downregulation of genes linked to structural developmental defects in congenital heart disease and to cholestasis in the hepatic network emphasizes the potential action of SBJ treatment to mitigate the identified gene transcripts. Interestingly, genes related to adult-onset myofibrillar myopathy 2A, a degenerative disease with progressive structural collapse of muscle fibers, were inhibited by the treatment. This downregulation of the muscular dystrophy network supports our findings in skeletal muscle PPI enrichment analysis showing activation of muscle fiber and regenerative network. Similarly, target tissues enriched from the AGR disease annotation overlapped with the tissue types that were highly enriched with upregulated gene sets. These patterns suggest a model wherein SBJ may exert a preferential targeted effect on these overlapping tissue types. This hypothesized mechanism is predicted to involve the promotion of lineage-specific cell growth and metabolic activation, alongside the concomitant downregulation of paths associated with pathological biological processes.

## 4. Discussion

In this study, we utilized a normal hAESCs spheroid culture model to investigate the primary effect of SBJ on baseline biological processes, leveraging its inherent multilineage potential, nontumorigenic profile, and low immunogenicity. Among the three dosage groups evaluated, treatment with 10 µg/mL SBJ resulted in a pronounced morphological expansion, showing larger spheroid diameter and surface area. Microarray analysis validated this spheroid structural growth by revealing the upregulation of key upstream markers of proliferation when the spheroids were treated with SBJ. Notably, the activation of downstream target genes regulated by E2F and MYC clearly indicates that the cells are primed for mitotic cell expansion to build the spheroid mass. Corroborating this G1/S phase priming, the G2M checkpoint hallmark pathway and biological process gene ontologies chromosome segregation (NES 1.35, *p* value = 0) and spindle assembly (NES 1.66, *p* value = 0) were significantly upregulated, demonstrating an active transition through the final stages of the cell cycle.

This SBJ-induced proliferation was supported by a uniform and coordinated upregulation of mitochondrial machinery, including oxidative phosphorylation, mitochondrial RNA processing, TCA cycle, and mitochondrial membrane organization-enriched processes. Interestingly, at the transcriptomic level, this metabolic activation occurs without the enrichment of reactive oxygen species (ROS) pathways or inflammatory signaling, as evidenced by a total lack of enrichment in TNFα via NF-κB and IL6/JAK/STAT3 signaling networks. While these computational profiles suggest a signature consistent with highly efficient and physiological metabolic activation rather than classical cellular stress or inflammatory responses, further direct phenotypic measurements of cellular ROS levels and inflammatory cytokines are required to conclusively confirm this lack of activation. Consistent with previous reports [7,44,50,51], this study demonstrates that the SBJ is rich in quercetin and isorhamnetin. While these major flavonoids likely contribute to the observed activation of mitochondrial respiration, they function within a highly complex, intact botanical matrix. Because we evaluated the whole juice extract rather than isolated fractions, these transcriptomic responses cannot be causally attributed to specific single compounds. Instead, it is highly probable that quercetin and isorhamnetin act in concert with other naturally occurring phytochemicals, nutrients, and co-factors present within the complete SBJ matrix to drive the observed cellular shifts. Taken together, the transcriptomic profiles suggest a signature consistent with highly efficient, physiological mitochondrial biogenesis and metabolic activation to fuel the accelerated proliferation, rather than classical cellular toxicity or mitochondrial stress pathways. However, because direct phenotypic measurements of cell viability, cytotoxicity, or apoptosis were not performed in this study, these transcriptomic-driven predictions require further functional validation to definitively rule out cellular toxicity.

GSEA initially indicated the upregulation of p53 pathway hallmark enrichment alongside the oxidative phosphorylation gene set. However, the *TP53* gene itself was significantly downregulated (fold change = −1.54, *p* value = 0.014), and canonical downstream transcriptional targets of p53, *MDM2*, *CDKN1A*, and *BAX*, were not present in either the DEG list or the GSEA leading-edge subset. This indicates that the observed enrichment of the p53 pathway was not driven by canonical p53 transcriptional activity. Instead, we attribute this enrichment to a passenger effect driven by the upregulation of overlapping metabolic transcripts that are shared by p53 and Oxidative phosphorylation hallmark gene sets [52]. Consequently, the observed phenotypic changes in SBJ-treated spheroids appear to be mediated by p53-independent metabolic reprogramming.

In the context of a 3D structural model, cells in the inner core, deprived of oxygen and nutrients, become hypoxic and metabolically switch to anaerobic glycolysis. In contrast, cells in the outer shell are exposed to a rich nutrient and oxygen environment that drives oxidative phosphorylation and proliferation. Accordingly, core hypoxia-inducible factor (HIF) target genes, including *SLC2A1* (glucose transporter), *PDK1* (pyruvate dehydrogenase kinase), *ADM* (stress adaptive factor), and *BNIP3L* (pro-apoptotic regulator), were identified among GSEA leading edge analysis of the hypoxia hallmark, confirming an active hypoxic survival program. Therefore, concurrent enrichment of oxidative phosphorylation, glycolysis, and hypoxia pathway hallmarks is an expected spatial gradient characteristic of healthy 3D spheroid growth and activated proliferation.

By evaluating the direct transcriptomic effect on normal hAESC spheroids, we sought to predict specific cell-like and tissue-like behaviors induced by the SBJ treatment. Our GSEA cell type signature analysis clearly revealed that the treated cells acquired highly proliferative and active ECM remodeling traits. When cross-referenced with the tissue type signature database, the cells shared strong transcriptional similarity with the proliferative state of the human embryo and Wharton’s jelly. Interestingly, across all cell type and tissue type annotation analyses, muscle type signatures were consistently enriched, including cardiomyocyte, myoblast, and smooth muscle lineages, indicating that the predicted signatures of SBJ treatment are predominantly enriched for broader mesodermal lineages, and more specifically for contractile and structural muscle types. Previous studies have established that sea buckthorn and its bioactive constituents mitigate cardiovascular disease by optimizing serum lipid profile with reduced triglycerides, cholesterol, and LDL [53,54] while suppressing inflammatory markers and ectopic fat accumulation [55]. Beyond these systemic metabolic improvements, sea buckthorn-derived extracts possess potent and localized anti-platelet and vasoprotective properties. For instance, preincubating freshly isolated human platelets with the extract directly decreased thrombin-activated platelet adhesion to both collagen and fibrinogen [56]. Vascular wall injuries expose underlying collagen fibers, which trigger platelet activation that can cause thrombosis and ischemic stroke if uncontrollably activated; therefore, the ability to modulate this cellular interaction is a critical therapeutic mechanism. Notably, this anti-platelet effect manifests prior to any detectable changes in body weight and plasma lipid profile in healthy individuals after four weeks of sea buckthorn oil supplementation [18]. Our transcriptomic findings generate a predictive hypothesis that bridges these established phenotypes with a potential cellular mechanism, establishing a foundation for future experimental validation. At the pathway level, the enrichment of artery-specific processes characterized by negative regulation of cell migration and small GTPase signaling loops points to a predicted footprint on both vascular endothelium and circulating platelets. These transcriptomic signatures suggest that by restricting aberrant smooth muscle cell migration and stabilizing small GTPase pathways, SBJ could potentially serve a protective role in modulating vascular remodeling and supporting endothelial integrity. Consistent with these data, treatment with sea buckthorn-derived procyanidins attenuated palmitic acid-induced oxidative damage in HUVECs by enhancing mitochondrial membrane potential increase and diminishing LDH leakage [57]. Furthermore, our transcriptomic profiling revealed a skin tissue enrichment signature paired with the downregulation of keratinization genes. When integrated with previous literature reporting the alleviation of vaginal atrophy [29], these computational alignments generate the hypothesis that the sea buckthorn may possess a putative role in counteracting pathways associated with premature senescence. The predicted mechanism suggests a potential capacity to support cellular plasticity and modulate uncommitted stem cell pools to favor tissue homeostasis.

Study limitations. While this study provides a comprehensive blueprint of the cellular responses to SBJ, several key limitations must be acknowledged. First, the primary differential expression mapping was exploratory in nature, utilizing unadjusted *p*-values to maximize screening sensitivity at the single-gene level. Second, while high-level functional analyses were maintained via strict FDR-corrected GSEA pipelines, these findings remain predictive. Direct phenotypic validation and functional assays such as direct cytotoxicity, cell viability, or tissue-specific regeneration measurements were not performed to physically verify the transcriptomic network models. Third, SBJ represents a complex botanical matrix inherently subject to lot-to-lot variations; although the two lots evaluated here showed distinct baseline concentrations of quercetin and isorhamnetin, their data were intentionally pooled to filter out batch-specific noise and isolate conserved biological signatures. Nevertheless, these compositional fluctuations remain a limitation, as variations in key phytochemicals could modulate the magnitude or kinetics of the cellular response. Finally, extreme caution must be exercised when extrapolating data from a single-donor in vitro 3D hAESC spheroid culture to specialized human tissues or complex systemic clinical outcomes. These data serve strictly as a hypothesis-generating, transcriptomic-based predictive baseline, highlighting the critical necessity for future in vivo disease models and controlled human bioequivalence evaluations.

Furthermore, we acknowledge as a limitation that a vehicle-matched DMSO control was not utilized for the control group cultures. While the final DMSO concentration in the highest treatment group was kept strictly at 0.1% (*v*/*v*), a level widely documented to minimize vehicle-induced artifacts or transcriptomic alterations [36], the potential, minor confounding influence of the vehicle cannot be completely ruled out. Future validation studies will incorporate parallel vehicle controls to definitively isolate the biological effects of the SBJ matrix.

## 5. Conclusions

In conclusion, through this descriptive microarray analysis, we mapped global gene expression in hEASC spheroids regulated by the SBJ to predict dynamic biological behaviors, signaling pathways, upstream regulators, and cell and tissue type signatures. Our analysis does not directly prove a physical outcome on its own, but it serves as a powerful tool to hypothesize how the tissue or cells respond to sea buckthorn intervention. The transcriptomic profiling predicts that SBJ potentially exhibits potent antioxidant activity across diverse tissue types, particularly epithelial and smooth muscle cells. This study demonstrates that evaluating the complete, non-fractionated matrix of freeze-dried SBJ induces robust mitochondrial biogenesis and metabolic activation in vitro. However, because these findings are restricted to a cell culture model utilizing a DMSO vehicle, direct human dietary recommendations cannot be derived from these data. Future in vivo evaluations, detailed bioavailability assessments, and mechanistic clinical studies are required to determine how these cellular benefits translate to human consumption and to evaluate the clinical efficacy of whole sea buckthorn juice as a practical dietary supplement or functional food.

## Data Availability

The microarray datasets generated in this study have been deposited in the NCBI Gene Expression Omnibus (GEO) and are accessible through accession number GSE342531.

## Supplementary Materials

The following supporting information can be downloaded at: https://www.mdpi.com/article/10.3390/nu18193285/s1, File S1: Complete list of DEGs.

## Abbreviations

The following abbreviations are used in this manuscript:

AGR	Alliance of Genome Resources
ARCHS4	All RNA-seq and ChIP-seq Sample and Signature Search
DEGs	Differentially Expressed Genes
DMSO	Dimethyl Sulfoxide
ECM	Extracellular Matrix
eNOS	endothelial Nitric Oxide Synthase
FDR	False Discovery Rate
GO	Gene Ontology
GSEA	Gene Set Enrichment Analysis
hAESCs	human Amniotic Epithelial Stem Cells
HPA	Human Protein Atlas
HPLC	High-Performance Liquid Chromatography
ICAM1	Intercellular Adhesion Molecule 1
IL6	interleukin-6
KEGG	Kyoto Encyclopedia of Genes and Genomes
LOX1	Lysyl Oxidase-Like 1
LPS	Lipopolysaccharide
NES	Normalized Enrichment Score
NF-κB	Nuclear Factor kappa-light-chain-enhancer of activated B cells
Nrf-2/HO1	Nuclear factor erythroid 2-related factor 2/Heme Oxygenase-1
PPI	Protein–Protein Interaction
SBJ	Sea Buckthorn Juice
TCA	Tricarboxylic Acid
TNFα	Tumor Necrosis Factor Alpha
